# A Large-Scale Study into Protist-Animal Interactions Based on Public Genomic Data Using DNA Barcodes

**DOI:** 10.3390/ani13142243

**Published:** 2023-07-08

**Authors:** Jiazheng Xie, Bowen Tan, Yi Zhang

**Affiliations:** Chongqing Key Laboratory of Big Data for Bio Intelligence, Chongqing University of Posts and Telecommunications, Chongqing 400065, China

**Keywords:** protist, DNA barcode, contamination, symbiosis, parasites, host-microbe interactions

## Abstract

**Simple Summary:**

Protists are a group of eukaryotic organisms that are important materials for studies of parasitology, metazoan/animal origin and mitochondrion evolution. However, as they are highly diverse and some species can infect animals with a broad host range, there is still a gap in knowledge regarding protist-animal interactions. Microbe contamination in genomic databases can not only confuse the results of genomic analysis but also provide valuable resources in research of microbe diversity and microbe-host interactions. In this study, we conducted a large-scale scan of protistan contaminations in a public genomic database based on DNA barcodes. The results suggest that there are high numbers of protistan contamination in animal assemblies in public genomic databases. And the heterogeneous distribution of protistan contaminations across different animal taxa reflects complicated protist-host relationships across different animal taxa.

**Abstract:**

With the birth of next-generation sequencing (NGS) technology, genomic data in public databases have increased exponentially. Unfortunately, exogenous contamination or intracellular parasite sequences in assemblies could confuse genomic analysis. Meanwhile, they can provide a valuable resource for studies of host-microbe interactions. Here, we used a strategy based on DNA barcodes to scan protistan contamination in the GenBank WGS/TSA database. The results showed a total of 13,952 metazoan/animal assemblies in GenBank, where 17,036 contigs were found to be protistan contaminants in 1507 assemblies (10.8%), with even higher contamination rates in taxa of Cnidaria (150/281), Crustacea (237/480), and Mollusca (107/410). Taxonomic analysis of the protists derived from these contigs showed variations in abundance and evenness of protistan contamination across different metazoan taxa, reflecting host preferences of Apicomplexa, Ciliophora, Oomycota and Symbiodiniaceae for mammals and birds, Crustacea, insects, and Cnidaria, respectively. Finally, mitochondrial proteins COX1 and CYTB were predicted from these contigs, and the phylogenetic analysis corroborated the protistan origination and heterogeneous distribution of the contaminated contigs. Overall, in this study, we conducted a large-scale scan of protistan contaminant in genomic resources, and the protistan sequences detected will help uncover the protist diversity and relationships of these picoeukaryotes with Metazoa.

## 1. Introduction

The advent of next-generation sequencing (NGS) technology has made genomic sequencing faster and cheaper. In recent years, the size of the public genomic database has skyrocketed. These data provide valuable resources for studies of genomic function and regulation of gene expression. However, contamination in the database may lead to spurious results [1]. Contamination could be generated in the library preparation, or caused by incidental infection or symbiosis of microbes [2,3]. Microbe contamination is a serious problem when the target DNA is a small amount, such as ancient DNA projects [4], but can also be a treasure trove of information for host-microbe interactions [5,6,7], especially when the microorganisms are difficult to isolate or culture. For example, a partial analysis of public databases found apicomplexan contaminations in 51 datasets across 920 assemblies [8].

However, contamination scanning is not a trivial thing because the genomic data deposited in public database can be quite large; systematic approaches to detect contamination therefore would be limited by computational costs, and cannot be undertaken without accounting for computational power [5,9]. Metabarcoding (DNA barcodes) is widely used to classify species in environmental microbial studies [10] and is occasionally applied to identify contamination. For example, DNA barcode COX1 was used to identify between-species contamination from the same laboratory’s co-occurrent transcriptome data [11]. The small size, reliability and robust ability of DNA barcodes to discriminate different species make them ideal markers to identify microbe contamination in huge genomic resources covering a broad range of animal species.

Protists are a group of highly diverse eukaryotic organisms that hold key roles in nearly all ecosystems [12,13]. Many protists are pathogenic parasites that can cause animal or plant diseases, as in the case of gregarines (Apicomplexa, Gregarinasina) in insects, coccidians (Apicomplexa, Eimeriidae) in mammals, and Oomycota (Stramenopiles) in plants [14,15]. Moreover, some protists are mutualistic, for example, Symbiodinium (Dinophyceae, Symbiodiniaceae) use light to produce photosynthate which is an essential food for coral (Cnidaria) [16]. And further, ciliate (Ciliophora) protozoans are one of the most diverse and frequent group of epibionts on the Crustacea species [17]. Apart from their symbiotic relation with Metazoa, they also provide important clues for research on the origin of Metazoa or multicellularity [18], such as how mitochondrial study of protists shed light on the mitochondrion evolution [19].

Here, to further our understanding of interactions between animals and the micro-eukaryotic protists, we conducted a systematic study of protistan contamination in metazoan assemblies using a strategy based on DNA barcodes. The following three questions are addressed: (i) Are the contamination rates different across different data types (WGS/TSA) or different animal taxa? (ii) How is the protistan contamination distributed among different animal taxa? Or who (animal host) is infected with what (protists)? (iii) How are these detected protists phylogenetically related to other known protists?

## 2. Materials and Methods

### 2.1. Database Retrieval

A total of 9487 WGS and 4465 TSA assemblies belonging to taxonomic groups of animals were downloaded from Genbank [20] (https://www.ncbi.nlm.nih.gov/Traces/wgs, accessed on 13 October 2022) (Spreadsheet S1). The total contig number is 1.489 billion, amounting to 11 trillion bp.

The Genbank nt database was downloaded from (https://ftp.ncbi.nlm.nih.gov/blast/db/, accessed on 27 December 2022).

The BOLD database, which is the largest DNA barcode reference library [21], was downloaded from (http://www.boldsystems.org/index.php/datapackages, version 03-Mar-2023, accessed on 5 March 2023). This package includes 9,253,201 DNA barcodes from 8,953,292 species.

### 2.2. Protistan Contamination Scanning Workflow

**Figure 1:**
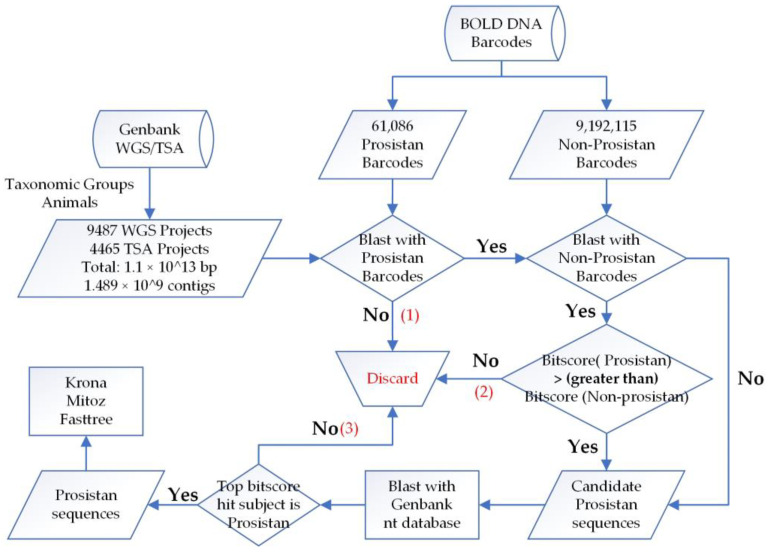
Overview of the bioinformatic pipeline used to scan protistan contamination. Three steps (1–3) reducing the amount of candidate sequences were marked red.

We used biopython [22] to deal with sequence format, and BLAST tool [23] to align sequences. As BLAST is computationally intensive, to overcome this shortage, we applied three steps to reduce the amount of candidate sequences (Figure 1):

First, the strategy of Platypus Conquistador [24], which uses inclusion and exclusion sets, was adopted. The BOLD database was divided into two sets: the protistan set for inclusion, with sequences of interest and in small volume (size: 57 Mb, number: 61,086), and the non-protistan set for exclusion (size: 6.5 Gb, number: 9,192,115). The contigs of assemblies were first blasted against the inclusion set to check if they were similar to protistan barcodes; only contigs with e-values < 1 × 10^−5^ were retained and the rest were discarded.

Next, candidate contigs were further blasted against the exclusion set (non-protistan barcodes), and the best score match was compared to that of the inclusion set. If there was no match with the exclusion set or the bitscore value of exclusion set was less than that of the inclusion set, that is, the contig showed more similarity to protist than non-protist, then it was retained for further analysis.

By searching against a small dataset (inclusion) first, and subsequently removing contigs with closer similarity to the exclusion set, the total candidate sequences were drastically reduced, therefore, affordable for alignment with the Genbank nt database to further reduce the false positive rate. Finally, if the corresponding subject of the best score match was of the protistan species (Section 3.2 presents additional decision steps in certain situations), then the contig aligned was classified as a protistan contaminant.

### 2.3. Taxonomic Analysis of the Protistan Contigs

Following the above verification from blasting against the nt database, the protist-contaminated contigs were assigned the taxonomic labels (taxids) of the corresponding subjects in the best score BLAST matches. The NCBI Taxonomy database [25] was used to identify the hierarchy of the taxonomic labels. Krona was used to estimate the abundance of protists across different metazoan taxa [26].

### 2.4. Phylogenetic Analysis

COX1 and CYTB proteins were predicted from the contaminated contigs with Mitoz [27] and aligned with MAFFT with maxiterate 1000 [28]. If multiple *CYTB* or *COX1* genes were predicted from a single assembly, we only chose the longest one for the evolutionary analysis. Next, a maximum-likelihood tree with a JTT+CAT model was inferred by FastTree with default parameters [29]. The resulting trees of both genes were rooted with the Choanoflagellata taxon *Monosiga brevicollis* and Ichthyosporea taxon *Sphaerothecum destruens*. All analyses were run on a dual Intel Xeon Platinum 8375C CPUs computer server.

## 3. Results

### 3.1. Classification of Protistan DNA Barcodes in the BOLD Database

**Figure 2:**
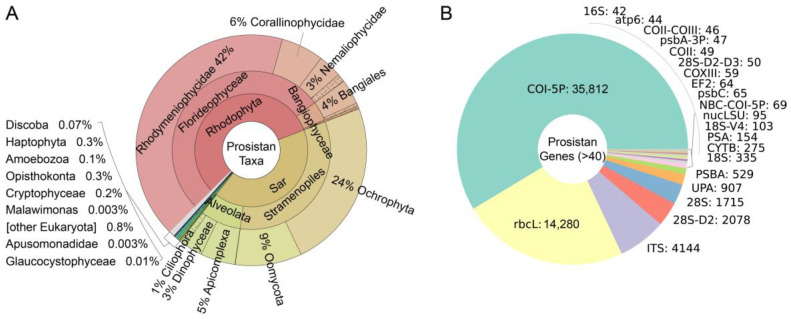
Distribution of DNA barcodes among taxa (percentage) (**A**) and genes (name: number) (**B**) of protists in BOLD database.

First, DNA barcodes from the BOLD database were divided into two groups: protistan (inclusion set) and non-protistan (exclusion set). The protistan set has 61,086 sequences, accounting for 0.66% of the total. To have a good understanding of these protistan DNA barcodes, we counted these barcodes by species (Figure 2A) or genes (Figure 2B). The results showed that most of these barcodes are from the Sar supergroup (41.5%) and Rhodophyta phylum (56.6%). The remaining about 2% of barcodes are from Haptophyta, Amoebozoa, Discoba, etc. As for the gene distribution, most are of mitochondrial gene *COI* (*COX1*) (58.6%) which has high accuracy in species assignment. The second most abundant gene is chloroplast *rbcL* (23.4%), then ITS (6.8%).

### 3.2. Protistan Contamination in the Genbank nt Database

Candidate contigs that have more similarity with the barcodes of protists than those of non-protists were selected and further blasted against the nt database to guarantee that all resulted contigs were truly protistan contamination.

When we carefully examined the blast results of candidate protistan contigs against the nt database, we found some sequences in nt database were wrongly annotated. To account for this problem, we adjusted our workflow after blasting against the nt database with an additional decision step: if the subject of best score match is a non-protistan sequence, but with 100% identity and same species to the assembly, then this alignment is possibly an annotated version of itself in the nt database and omitted, and the next best score alignment will be checked recursively. If the next alignment is a protistan subject, the contig and the previous nt subject will be classified as protistan contaminants. In this way, we found a dozen mis-annotated sequences that are actually protistan contaminants in the Genbank nt database (Table 1). Notably, XM_015829859.1 and XM_015829860.1 were mis-annotated to *COX1*/*CYTB* like genes of *Protobothrops mucrosquamatus* (snake), but actually are of Coccidia (Apicomplexa); XR_003895254.1-XR_003895257.1 are from *Aedes albopictus* (mosquito), but actually are of Conoidasida (Apicomplexa).

### 3.3. Heterogeneous Contamination Rates across Different Animal Taxa

In this subsection, we analyzed the number of protist-contaminated assemblies among different data types (WGS/TSA) or animal taxa. A total of 13,952 assemblies, including 9487 WGS (68%) and 4465 TSA (32%) in Genbank, were scanned. Protistan contamination was detected in 1507 assemblies (408 WGS and 1099 TSA). Thus, the TSA assemblies (24.6%) are more prone to protistan contamination than WGS assemblies (4.3%).

**Figure 3:**
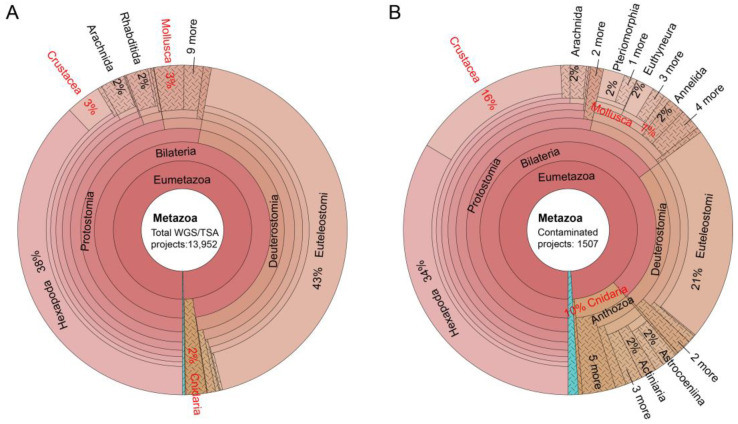
Krona plot illustrating numbers of total (**A**) and protist-contaminated (**B**) metazoan assemblies/projects at various metazoan taxonomic levels. Three animal taxa with significantly higher contamination rates were marked in red font.

We next inspected the protistan contamination across different animal taxa, and found heterogeneous contamination rates across different animal taxa. For example, the assembly numbers of Mollusca, Crustacea and Cnidaria are 410, 480 and 281, representing 3%, 3% and 2% of the total 13,952 assemblies/projects, respectively (Figure 3A). While among the found 1507 contaminated assemblies, there are 107 Mollusca, 237 Crustacea and 150 Cnidaria assemblies, amounting to 7%, 16% and 10% of total contaminated assemblies, respectively (Figure 3B). Thus, contamination rates (26%, 49% and 53%) in these three taxa are significantly higher than average 1507/13,952 (11%). We also inspected contamination rates of other animal taxa and the results are below: 67/2689 for Mammalia, 75/925 for Aves (birds), 117/1944 for Actinopterygii (bony fishes), and 506/5308 for Hexapoda (insects). The contamination therefore is heterogeneous, reflecting various protist-host relationships across different animal taxa.

### 3.4. Protistan Contamination Is Host Species-Specific

We next investigated the number and source species of the protist-contaminated contigs detected. A total of 17,036 protistan contigs were detected in the GenBank WGS/TSA database (Spreadsheet S2 & Fastafile S1). Most of these are of the Sar supergroup (13,531), followed by Rhodophyta phylum (1303) (Figure S1A).

To further explore the heterogeneous distribution of the protistan contamination, we compared the relative abundance of contaminated contigs at various protistan taxonomic levels across different animal taxa (Figure S1B–F). At first, we found the major phylogenetic units causing the contamination were different (Table S1). For instance, the dominant clades of Stramenopiles and Alveolata on average were Ochrophyta and Ciliophora, respectively, while the dominant phylum of Stramenopiles in insects was Oomycota (317/563). Among them, the majority were Peronosporaceae (138) and Albugo (144), which are among the top oomycete pathogens of plants [30]. We suppose these Oomycota species were likely transferred from plants to insects during feeding. Additionally, the dominant phyla of Alveolata in mammals and Cnidaria were Apicomplexa (246/260) and dinoflagellates (1609/2181), respectively. Among these dinoflagellates, Symbiodiniaceae (1368/1609) were in the majority. This likely reflects the symbiotic relationships of dinoflagellates with Cnidaria [31]. Although the majority of Apicomplexa in mammals and birds were both Eimeriorina (Coccidia), the second most abundant taxa were different, with Haemosporida in birds and Piroplasmida in mammals (Figure S1E,F).

**Figure 4:**
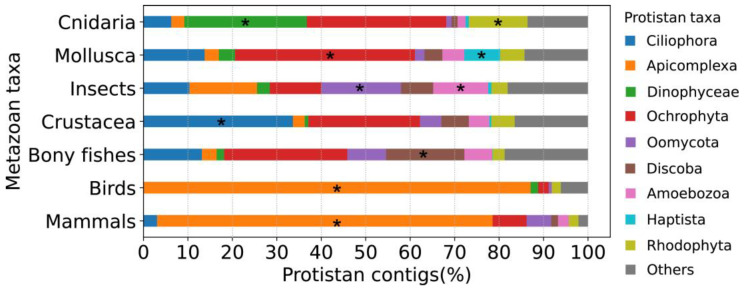
Distribution patterns of protist-contaminated contigs in assemblies of different animal taxa. The percentages of contigs belonging to each protistan taxon were calculated in the following way, taking Apicomplexa in mammals as an example: ‘Number of Apicomplexa-contaminated contigs in mammal assemblies’/‘Number of all protist-contaminated contigs in mammal assemblies’. Asterisks (*) denote the most representative animal taxa of each protistan taxon.

Based on the above host species-specific distribution, we further calculated the relative abundance of contaminated contigs belonging to different protistan taxa in the following metazoan taxa: mammals, birds, bony fishes, Crustacea, insects, Mollusca and Cnidaria (Figure 4 and Table S2). The results were consistent with the above observation that different metazoan taxa have a different distribution of protists. For example, percentages of contaminated contigs belonging to Oomycota, Apicomplexa and Dinophyceae were higher in insects, mammals/birds and Cnidaria, respectively.

### 3.5. Evolutionary Analysis of the Contamination Contigs

**Figure 5:**
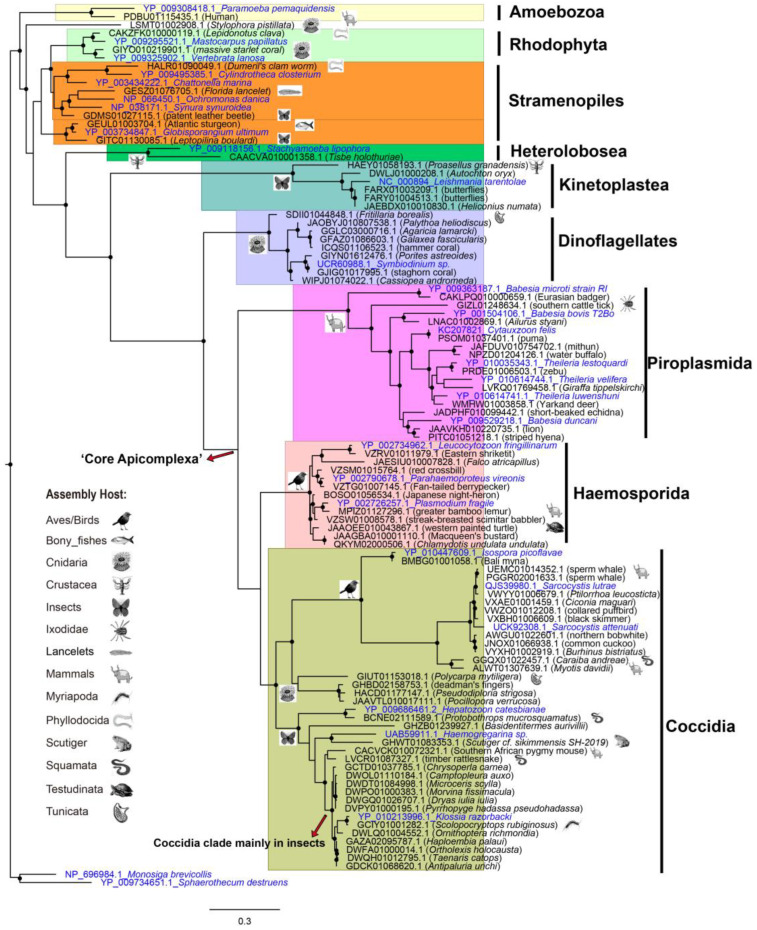
Maximum-likelihood tree of COX1 predicted from contaminated contigs found in WGS/TSA assemblies, with *Monosiga brevicollis* (Choanoflagellata) and *Sphaerothecum destruens* (Ichthyosporea) as outgroups. The source species of the assemblies are marked by animal symbols. Nodes with more than 50% branches of a single animal taxon are marked with the corresponding symbols, with the exceptional branches marked individually. Nodes with a bootstrap larger than 70% are denoted by a dark circle. The predicted COX1 was named with the accession number and species names (animal) of the source contigs, while the references retrieved from GenBank were colored in blue and named in the format of ‘accession number_species name (protist)’.

To understand the phylogenetic origin of the protists derived from these contamination contigs, we predicted the mitochondrial genes with Mitoz, and constructed phylogenetic trees with the predicted COX1 (Figure 5) and CYTB (Figure S2). As many protists have lost *CYTB* and *COX1* genes [32], we only collected 78 assemblies that have both predicted CYTB and COX1 longer than 80 amino acids. Among this smaller dataset, there exists previously described Sarcocystis (Coccidia) contamination in the assemblies of sperm whale (UEMC01 and PGGR02), northern bobwhite (AWGU01) and *Myotis davidii* (ALWT01) [33,34]. Furthermore, almost the same tree topology is observed for predicted COX1 and CYTB, corroborating the protistan origination of these contaminated contigs.

As the preceding subsection revealed, host-specific protist distribution is also observed in the phylogenetic tree resulting from the smaller dataset. For example, assemblies of mammals and birds have an abundance of apicomplexan contamination, with the order Piroplasmida most represented by mammals, and Haemosporida by birds. Haemosporida are globally distributed and can cause malaria-like diseases in birds [35]. In addition, the clade of dinoflagellates is mainly composed of protists derived from contigs of Cnidaria.

Interestingly, there was a large amount of Kinetoplastea (kinetoplasts) and Coccidia in insect assemblies. This observation is consistent with the study of protozoa which showed that amoebas, coccidia and kinetoplastids were among the main taxa observed in the model insect *Nauphoeta cinerea* [36].

Coccidia are underestimated parasites of the Insecta, and have very limited species definitions except the genus Adelina [37]. However, contaminants of Coccidia in insects detected in this study included the genus of Adelina (31 contigs), Klossia (71 contigs) and Eimeriorina (54 contigs). Thus, we suppose that Coccidia are common in Insecta and need more study.

In addition, we also observed sporadic protists within some animal taxa, such as Piroplasmida in ticks (Ixodidae) (GIZL01), Haemosporida in turtles (Testudinata) (JAAOEE01), and Coccidia in toads (Scutiger) (GHWT01), snakes (Squamata, Serpentes) (GGQX01, BCNE02 and LVCR01) and centipedes (Myriapoda, Chilopoda) (GCIY01). Among these protist lineages, Hemosporidian parasites in turtles and Cyclospora (Coccidia, Eimeriidae) in snakes and Glomeris (Myriapoda, Diplopoda) have been described [38,39]. However, no Coccidia in centipedes has been reported to date. To our knowledge, this finding of Coccidia in *Scolopocryptops rubiginosus* (GCIY01) is the first reported case of Coccidia in centipedes.

Here we also observed the unusual clade of Coccidia in Cnidaria. We further checked source contigs of this lineage, and found that all three contigs of GHBD02158753.1, HACD01177147.1, and JAAVTL010017111.1 were blasted with MH320093.1 (Spreadsheet S2) from *Apicomplexa* sp. *WK-2018_Corallicola*, described in the paper as “A widespread coral-infecting apicomplexan with chlorophyll biosynthesis genes” [40]. This unusual clade indicates these photosynthetic relatives of apicomplexans are abundant in Cnidaria.

## 4. Discussion

DNA barcodes which have highly variable regions are currently the most effective ‘markers’ for species identification [41]. This study exploited this attribute to scan protistan contamination and detected 17,036 protist contaminated contigs in Genbank WGS/TSA assemblies. The identity of the best bitscore match in alignments of these contigs with the nt database is between 0.71 to 1, with an average of 0.93 and median of 0.95. There are 771 contigs with an identity less than 0.8, and 3756 contigs with an identity between 0.8–0.9. Thus, many of the contigs detected are from novel protists. As protists are often neglected or overlooked in the study of microorganisms, these protistan contigs provide a valuable resource for studies of diversity of protists.

We would like to emphasize, however, that the goal of this study was not to find all the protistan contaminants in the genomic data, but limited to finding contaminants related to DNA barcodes at a relatively affordable computing resource. Considering the large and rapidly growing number of WGS/TSA assemblies, detecting all the contaminants related to all protistan genes in the whole WGS/TSA database would be beyond the acceptable computational power, especially if the candidate contigs need to be further aligned to the Genbank nt database to minimize the false positive rate. However, as the DNA barcodes are mostly located in mitochondrial or rRNA genes which are multi-copy and high-expressed, this study found contaminated assemblies of acceptable sensitivity. In addition, most of the output contigs are mitochondrial or rRNA sequences, and are therefore appropriate for subsequent evolutionary analysis.

The strategy of detecting hidden contaminants related to particular ‘marker genes’ in a public database has occasionally been reported, such as the detection of insect contamination by odorant-binding proteins (OBPs) and chemosensory proteins (CSPs) in plant transcriptomes [42], and searching apicomplexan parasite in animals using apicortin protein [33]. Here, we scanned protistan contamination related to the ‘markers’ of DNA barcodes, and revealed the pattern of host-specific contamination based on the output contigs.

Finally, *CYTB* and *COX1* genes were predicted and used for evolutionary analysis. However, there are still many unexplored output contigs for the following reasons: first, some contigs are predicted with a CYTB/COX1 length less than 80 amino acids and thus omitted for subsequent evolutionary analysis; second, a large proportion of contigs are rRNA or chloroplast genes, and thus have no CYTB/COX1 predicted, such as contigs belonging to taxon of Ochrophyta (5173 out of 6115 contigs), amitochondriate protist Metamonada (all 129 contigs) and Archamoebae (all 121 contigs) (Figure S3).

## 5. Conclusions

The development of NGS technology has resulted in a tremendous growth of genomic data in public databases. The intrinsic microbial sequences provide good material for studies of host-microbe interactions. DNA barcodes are broadly used to study microbiology diversity in metabarcoding experiments, but are rarely used in database analysis by bioinformatics methods.

In this study, we present a bioinformatic pipeline to scan contaminants related to DNA barcodes in animal assemblies from Genbank at a relatively affordable computing resource cost. Based on these protistan contigs, we conducted a large-scale study of the distribution pattern of protists across different metazoan taxa. The results showed that about one in ten of metazoan assemblies is contaminated by protists, with even higher rates in assemblies of Mollusca, Crustacea and Cnidaria. Raising awareness about the widespread contamination in public genomic databases, especially transcriptome database, will help avoid misleading results. Interestingly, the contamination pattern is host species-specific, with higher relative abundance of contaminants belonging to Apicomplexa, Oomycota, Ciliophora and Symbiodiniaceae in Amniota (mammals and birds), insects, Crustacea and Cnidaria, respectively. The results are compatible with the relationships of Metazoa-Protists concluded in traditional studies. Thus, our pipeline is a reliable approach for host-microbe study based on the detected contaminant in public databases. Overall, our study provides valuable insights into the parasitic or mutualistic relationships between multicellular animals and the unicellular protists.

## Figures and Tables

**Table 1:** Protistan contamination in the Genbank nt database.

Mis-Annotated ^1^	Nt Subject (% Identity)	Len ^2^	Subject Title ^3^
XM_005911631.1	XM_005911631.1 (100)	999	*Bos mutus COX1*-like (LOC102267288)
	KT901048.1 (100)	633	*Sarcocystis hirsuta* isolate B10.7 (*COX1*) gene
XM_009575503.1	XM_009575503.1 (100)	1469	*Fulmarus glacialis COX1*-like (LOC104075110)
	LC602189.1 (86)	908	*Hyaloklossia kasumiensis* p01 mitochondrial *COX1* gene
XM_049994226.1	XM_049994226.1 (100)	5365	*Schistocerca gregaria* uncharacterized LOC126321793
	GU828005.1 (78)	1107	*Hartmannella vermiformis* mitochondrion, complete genome
XM_014483700.1	XM_014483700.1 (100)	996	*Bos mutus COX1*-like (LOC102278784)
	LC481080.1 (88)	666	*Sarcocystis pilosa* E044-4 mitochondrial *COX1* gene
XM_022922694.1	XM_022922694.1 (100)	1719	*Stylophora pistillata COX1*-like (LOC111319986)
	KU164874.1 (73)	1204	*Pleurocladia lacustris* strain SAG 25.93 mitochondrion genome
XM_015829859.1	XM_015829859.1 (100)	1143	*Protobothrops mucrosquamatus COX1*-like (LOC107300543)
	MK452388.1 (86)	1084	*Hepatozoon griseisciuri* genotype A mitochondrion genome
XM_015829860.1	XM_015829860.1 (100)	1126	*Protobothrops mucrosquamatus CYTB*-like (LOC107300544)
	MT936931.1 (85)	1048	*Hepatozoon* sp. mitochondrion, complete genome
XR_003895254.1- XR_003895257.1 ^4^	XR_003895257.1 (100)	3270	*Aedes albopictus* large subunit rRNA (LOC115262384)
	EF666482.1 (99)	2743	*Ascogregarina taiwanensis* 18S rRNA gene
XM_006777742.1	XM_006777742.1 (100)	1170	*Myotis davidii COX1*-like (LOC102771221)
	KT363924.1 (87)	1172	*Toxoplasma gondii* strain GT1 (*COX1*) gene
XM_009556771.1	XM_009556771.1 (100)	864	*Cuculus canorus CYTB*-like (LOC104055630)
	OK001464.1 (95)	859	*Sarcocystis* sp. JHu-2021a isolate Sarsa4 (*CYTB*) gene

^1^ The contamination sequences in the Genbank nt database were aligned to nt database for confirmation, the top two alignments with highest bitscore were listed. And the first record was alignment with itself. ^2^ Alignment length. ^3^ Abbreviation: ‘cytochrome b’: *CYTB*; ‘cytochrome c oxidase subunit 1’: *COX1*; ‘ribosomal RNA’: rRNA. ^4^ XR_003895254.1-XR_003895257.1 are 99.9% identical.

## Data Availability

The animal WGS/TSA datasets for this study can be found in [GenBank] (https://www.ncbi.nlm.nih.gov/GenBank, accessed on 13 October 2022). The bold DNA barcode library can be found in [Bold] (http://www.boldsystems.org/index.php/datapackages, accessed on 5 March 2023). The bioinformatic code is available at (https://github.com/xiebio/DBCscan, accessed on 3 April 2023).

## Supplementary Materials

The following supporting information can be downloaded at: https://www.mdpi.com/article/10.3390/ani13142243/s1, Figure S1: Relative abundance of protistan contigs at various metazoan taxonomic levels detected in the assemblies of all Metazoa (A) and different sub-taxa: insects (B), Mollusca (C), Cnidaria (D), mammals (E), and Aves (F); Figure S2: Maximum-likelihood tree of CYTB predicted from protist-contaminated contigs; Figure S3: Krona plot of contaminated contigs belonging to amitochondriate protist Metamonada and Archamoebae in WGS/TSA assemblies; Table S1: Different major phylogenetic units causing the contamination in different animal taxa; Table S2: Number of contigs belonging to different protistan taxa in assemblies of following animal species taxa: mammals, birds, bony fishes, Crustacea, insects, Mollusca and Cnidaria; Spreadsheet S1: WGS & TSA assembly info; Spreadsheet S2: Contamination contigs VS nt; Fastafile S1: Prosist contaminated contigs.fasta.
