# Supplementary material for: A Large-Scale Study into Protist-Animal Interactions Based on Public Genomic Data Using DNA Barcodes
Source: Animals (Basel). 2023 Jul 8;13(14):2243. doi: 10.3390/ani13142243 (PMC10376638; doi:10.3390/ani13142243)
Supplement: Supplementary file 1 [file animals-13-02243-s001.zip › Supplementary files/Supplementary figure and tables.pdf]

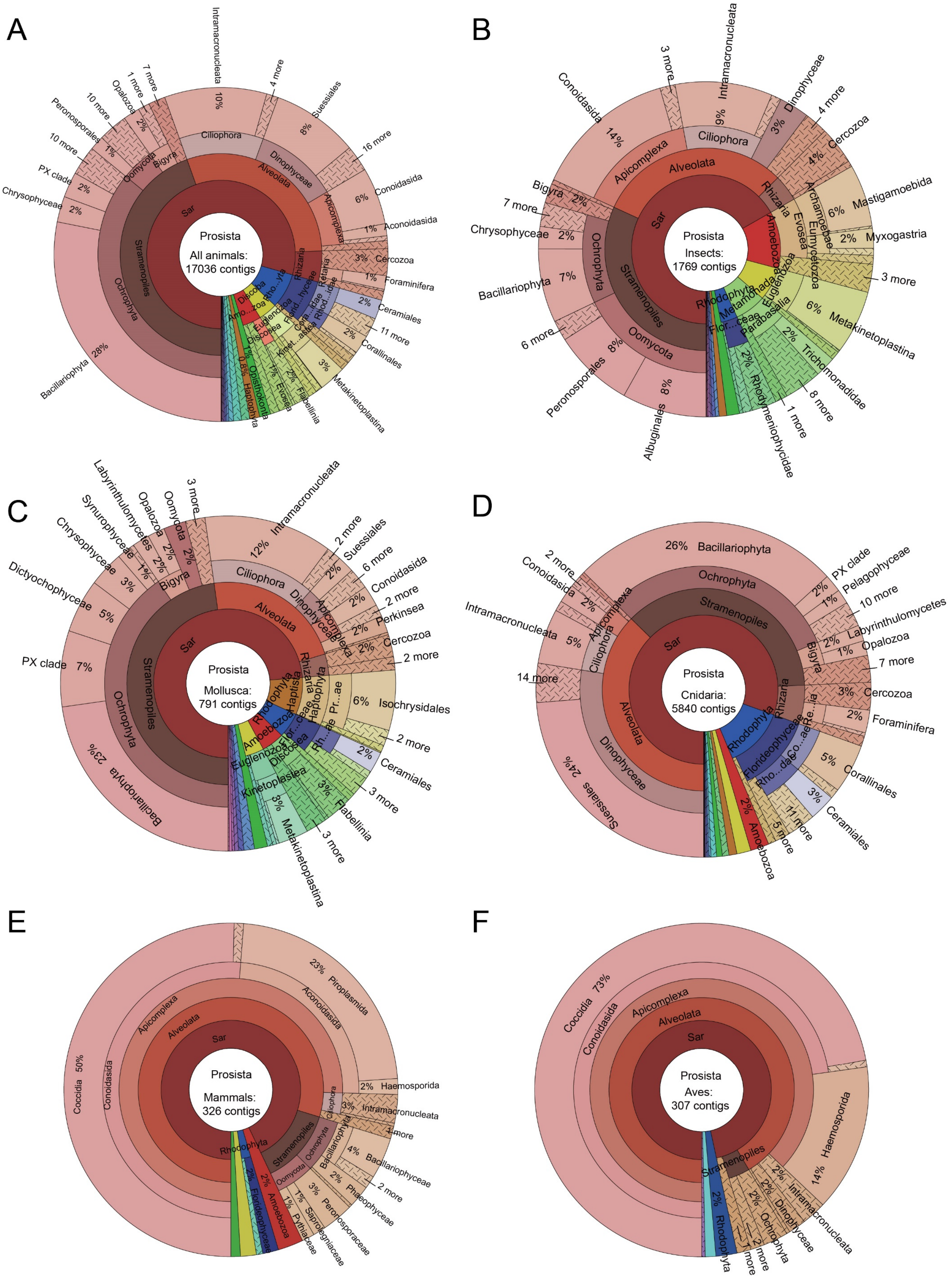


**Figure S1.** Relative abundance of protistan contigs at various metazoan taxonomic levels detected in the assemblies of all Metazoa (A) and different sub-taxa: insects (B), Mollusca(C), Cnidaria (D), Mammals (E), and Aves (F).

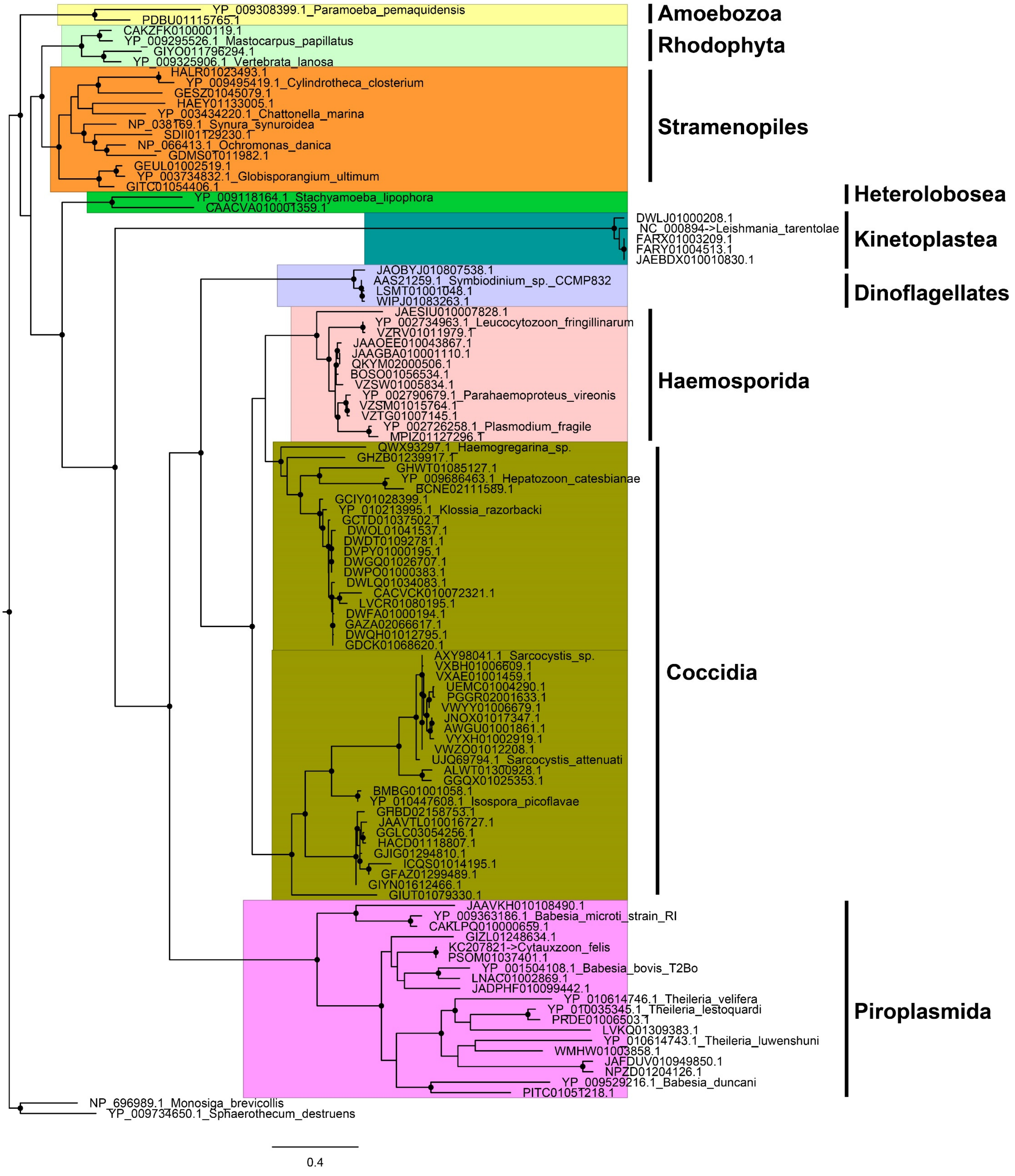


**Figure S2.** Maximum likelihood tree of CYTB predicted from protist-contaminated contigs. Nodes with bootstrap larger than 70% are denoted by a dark circle. The tree was rooted with the Choanoflagellata taxon *Monosiga brevicollis* and Ichthyosporea taxon *Sphaerothecum destruens*. The reference sequences are named in the format of 'Accession number_species name'.

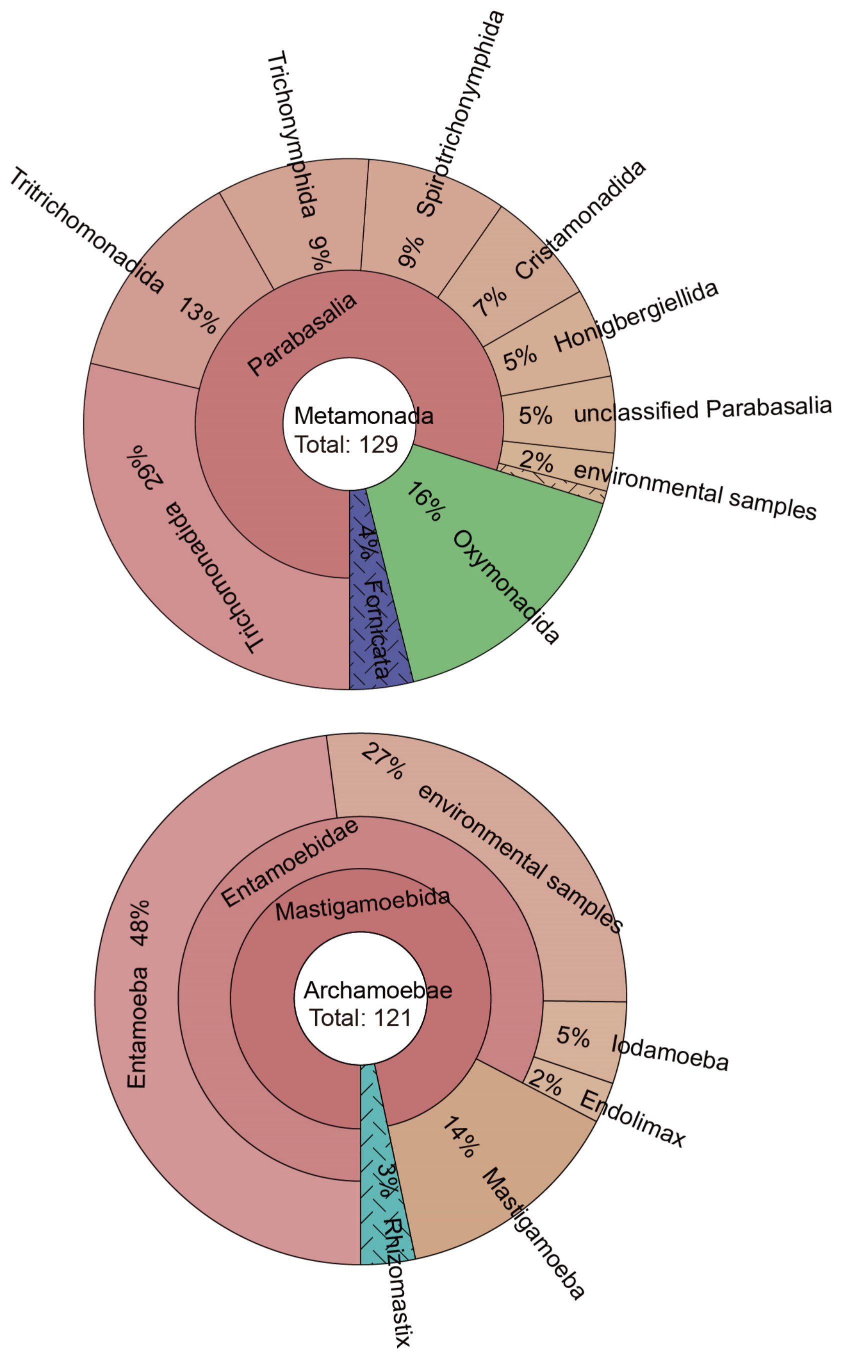


**Figure S3.** Krona plot of contaminated contigs belonging to amitochondriate protist Metamonada and Archamoebae in WGS/TSA assemblies.

**Table S1.** Different major phylogenetic units causing the contamination in different animal taxa. The ratios were calculated by dividing the number of contaminated contigs in each protistan taxa by that of the corresponding parent node.

| Phylogenetic unit | | Mammals | Bony fishes | Crustacea | Insects | Mollusca | Cnidaria |
|---|---|---|---|---|---|---|---|
| Alveolata | Ciliophora | 10/260 | 143/200 | 657/740 | 183/543 | 109/178 | 367/2181 |
| | Apicomplexa | 246/260 | 36/200 | 52/740 | 269/543 | 25/178 | 170/2181 |
| | dinoflagellates | 0 | 18/200 | 16/740 | 51/543 | 29/178 | 1609/2181 |
| Stramenopiles | Ochrophyta | 25/43 | 302/441 | 490/715 | 205/563 | 320/378 | 1834/2158 |
| | Oomycota | 18/43 | 94/441 | 95/715 | 317/563 | 17/378 | 68/2158 |

**Table S2.** Number of contigs belonging to different protistan taxa in assemblies of following animal species taxa: mammals, birds, bony fishes, Crustacea, insects, Mollusca and Cnidaria.

| Protistan taxa | Mammals | Aves | Bony fishes | Crustacea | Insects | Mollusca | Cnidaria |
|---|---|---|---|---|---|---|---|
| Ciliophora | 10 | 0 | 143 | 657 | 183 | 109 | 367 |
| Apicomplexa | 246 | 269 | 36 | 52 | 269 | 25 | 170 |
| Dinophyceae | 0 | 5 | 18 | 16 | 51 | 29 | 1609 |
| Ochrophyta | 25 | 7 | 302 | 490 | 205 | 320 | 1834 |
| Oomycota | 18 | 2 | 94 | 95 | 317 | 17 | 68 |
| Discoba | 5 | 0 | 192 | 120 | 128 | 32 | 79 |
| Amoebozoa | 8 | 0 | 68 | 90 | 219 | 39 | 105 |
| Haptista | 0 | 0 | 1 | 9 | 13 | 64 | 45 |
| Rhodophyta | 7 | 6 | 29 | 102 | 64 | 43 | 769 |
| Others | 7 | 18 | 204 | 322 | 320 | 113 | 794 |
| Total contaminated contigs | 326 | 307 | 1087 | 1953 | 1769 | 791 | 5840 |
